# Selective Conversion of Glycerol to Methanol over CaO-Modified HZSM-5 Zeolite

**DOI:** 10.3390/molecules27217221

**Published:** 2022-10-25

**Authors:** Thachapan Atchimarungsri, Xinhua Gao, Kangzhou Wang, Qingxiang Ma, Jianli Zhang, Subing Fan, Fugui He, Jumei Tian, Prasert Reubroycharoen, Tiansheng Zhao

**Affiliations:** 1 State Key Laboratory of High-Efficiency Utilization of Coal and Green Chemical Engineering, College of Chemistry & Chemical Engineering, Ningxia University, Yinchuan 750021, China; 2 Department of Chemical Technology, Faculty of Science, Chulalongkorn University, Bangkok 10330, Thailand; 3 Ningxia Academy of Metrology & Quality Inspection, National Quality Supervision and Inspection Center for Coal and Coal Chemical Products (Ningxia), Yinchuan 750200, China

**Keywords:** glycerol, methanol, HZSM-5, CaO

## Abstract

Biodiesel is generally produced from vegetable oils and methanol, which also generates glycerol as byproduct. To improve the overall economic performance of the process, the selective formation of methanol from glycerol is important in biodiesel production. In the present study, a CaO modified HZSM-5 zeolite was prepared by an impregnation method and used for the conversion of glycerol to methanol. We found that the 10%CaO/HZSM-5 with Si/Al ratio of 38 exhibited highest selectivity to methanol of 70%, with a glycerol conversion of 100% under 340 ℃ and atmospheric pressure. The characterization results showed that the introduction of a small amount of CaO into the HZSM-5 did not affect the structure of zeolite. The incorporation of HZSM-5 as an acidic catalyst and CaO as a basic catalyst in a synergistic catalysis system led to higher conversion of glycerol and selectivity of methanol.

## 1. Introduction

Recently, demand for biosources of energy-related compounds has been increasing, owing to reduced consumption of fossil sources. Biofuel is being recognized as an alternate for sustainable energy production. Biodiesel is an important biofuel, as it is now used as a substitute of the petroleum-based fuels. However, about 10 wt% of glycerol is produced as a by-product during transesterification reactions in biodiesel production [1,2]. Therefore, the utilization of glycerol has become an essential issue to produce various value-added chemicals and fuels [3,4,5]. Among them, the catalytic conversion of glycerol to methanol is attracting increasing attention.

Biodiesel is generally produced from the transesterification of triglycerides with methanol. Therefore, the transformation of glycerol to methanol would be highly desirable. This would be a good way to make the biodiesel process more sustainable, without using methanol derived from fossil sources. Also, methanol is an important chemical for producing hydrocarbon fuels and other industrial chemicals such as formaldehyde, acetic acid, and dimethyl ether [6,7,8]. Moreover, the methanol produced from a biomass feedstock is a sustainable alternative with economic and environment benefits for biodiesel production. There are a few heterogeneous catalysts for hydrogenolysis reaction of glycerol to methanol, such as Ni, Re, Cu, Zn, Mo, W, and V, supported on SiO_2_ or Al_2_O_3_ [9,10,11,12,13]. However, reactions over these catalysts are generally carried out at high temperature and/or pressure with the addition of H_2_ or O_2_ gases [9,10,11,12,13,14]. On the other hand, many researchers have opted to use autoclave batch reactors for these reactions [15,16]. These systems are often limited by problems such as the use of toxic metals, low yields, harsh reaction conditions, and the need to perform purification procedures. Thus, it is necessary to develop a facile and green route to directly convert glycerol to methanol under mild conditions in a fixed bed flow reactor. Hutching et al. [5] noted that glycerol can be reacted with water to form methanol without the addition of H_2_ gas in a low-pressure process. They used a basic or redox oxide, such as CaO, MgO, or CeO_2_, as a catalyst. However, the conversion of glycerol was low, leading to a poor efficiency. Kim et al. [17] studied the effect of Si/Al ratio in ZSM-5 on the glycerol conversion to acrolein. They found that the number of acid sites and the acid strength of ZSM-5 had a great influence on the catalytic activity and product selectivity. However, acrolein is currently produced as the main product over these solid acid zeolite catalysts. Thus, we propose that a combination of basic catalysts such as CaO and HZSM-5 is a promising method to enhance the catalytic activity and product selectivity to methanol.

In this work, we present a bi-functional CaO/HZSM-5 catalyst with high performance in the hydrogenolysis of glycerol to methanol. The influences of the CaO loading amount, the Si/Al ratio of the HZSM-5, WHSV, and the reaction temperature in this reaction are investigated for the conversion of glycerol to methanol.

## 2. Experimental

### 2.1. Preparation of Catalyst

#### CaO/HZSM-5 Catalysts Preparation

Commercial HZSM-5 was purchased from the Nanjing XF NANO, China, and was modified by CaO species via the wet impregnation method. HZSM-5 zeolites were added to the calcium nitrate tetrahydrate solution under stirring for 2 h. The samples were dried at 110 °C overnight, calcined at 550 °C at a heating rate of 10 °C min^−1^, and held at that temperature for 3 h. The amount of CaO loading was 0, 5, 10, and 20 wt%. All samples were pressed, crushed, and sieved to sizes of between 20–40 mesh before use.

### 2.2. Characterizations

Powder X-ray diffraction (XRD) patterns for all catalysts were obtained on a Rigaku D/MAX2200PC instrument with Cu Kα radiation (λ = 0.154 nm) at 40 kV and 40 mA. The N_2_ adsorption–desorption isotherms of the catalysts were measured with an Autosorb-iQ apparatus at 77K. A high-resolution scanning transmission electron microscope (HR-STEM) and high-angle annular dark-field scanning transmission electron microscope (HAADF-STEM) were used, and EDS mapping was conducted with a FEI Talos 200X. The acidity and basicity of the catalyst samples were measured by NH_3_-TPD and CO_2_-TPD experiments, respectively. The acid types of the catalysts were measured by pyridine adsorption FT-IR spectroscopy using a Bruker Tensor 27 instrument attached to an in situ cell. Thermogravimetric analysis (TGA) of the spent samples was conducted on a NETZSCH STA 449 F5.

### 2.3. Catalyst Performance

The conversion of glycerol into methanol was tested in a fixed-bed reactor under atmospheric pressure at 300–380 °C. To generate gaseous stream with 15 wt% of glycerol in the aqueous phase, a glycerol solution was injected into the oven and preheater at temperatures of 180 °C and 290 °C, respectively, by a high-pressure pump. The glycerol vapor was carried with a N2 flow of 20 mL·min^−1^. The catalyst sample was placed into a stainless steel tube with a 5 mm inner diameter and held in place between two layers of quartz wool. A thermocouple was placed in the catalyst bed and used to control the reaction temperature. The liquid products were collected for analysis after a reaction time of 2 h. The products were collected using an ice-water trap and analyzed with gas chromatography, coupled with a flame ionization detector (FID) and a DB-WAX Ultra Inert capillary column (30 m × 0.25 mm id, film thickness 0.25 µ) using 1-butanol as an internal standard.

The conversion of glycerol and product selectivity was calculated using the following equations: 

Glycerol conversion (%):

(1) $$X=\frac{n_{gly}^{Input}-n_{gly}^{Output}}{n_{gly}^{Input}}\times 100\%$$

Product *i* selectivity (%):

(2) $$S=\frac{n_{i}}{\sum n_{i}}\times 100\%$$

where $n_{i}$ is the mole of product *i* at the liquid outlet.

## 3. Results and Discussion

### 3.1. Characterization of the Catalysts

**Figure 1:**
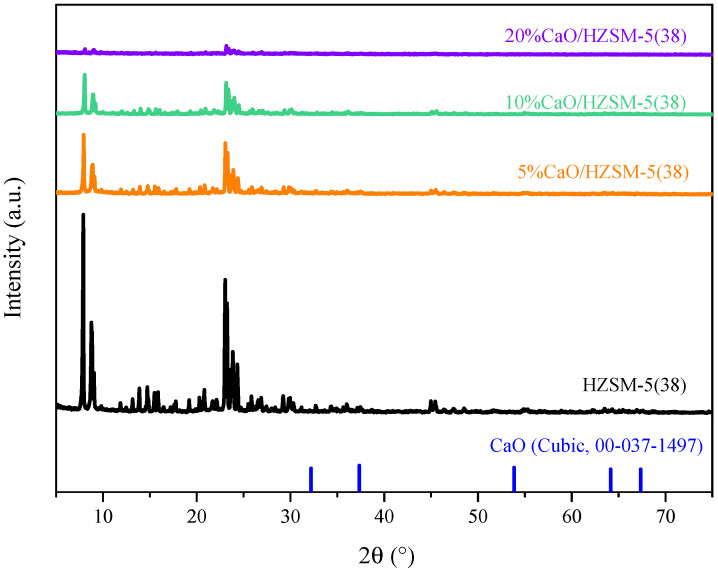
XRD patterns within the 2θ range of 5° to 60° of 5%, 10% and 20% of CaO loading over HZSM-5(38) catalysts.

Figure 1 shows the XRD patterns of CaO supported over HZSM-5(38) zeolites with different CaO loading contents. The diffraction peaks in the 2θ of 7°–25° corresponded to the typical formation of MFI structures of HZSM-5 for all sample powders [18]. Although the amount of CaO varied from 5% to 20%, the XRD patterns of the diffraction peaks of CaO modified HZSM-5 show a similar XRD pattern to the original HZSM-5, and there was no peak showing impurities in the CaO/ZSM-5 samples. It was confirmed that the CaO species were uniformly dispersed on the external surface of the zeolites [19]. Moreover, the intensities of the diffraction peaks of the modified HZSM-5 catalysts decreased in comparison with those of the original HZSM-5, which may have indicated the zeolite structure had been partially destroyed after CaO impregnation [20,21].

**Figure 2:**
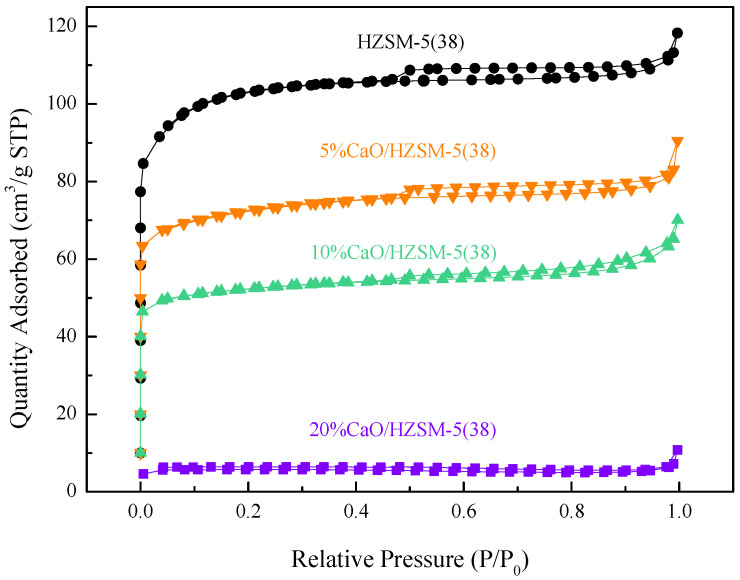
N_2_ adsorption–desorption isotherms of the 20%CaO/HZSM-5(38), 10%CaO/HZSM-5(38), 5%CaO/HZSM-5(38) and HZSM-5(38) catalysts.

Figure 2 presents the N_2_ adsorption–desorption isotherms of the HZSM-5(38), 5%CaO/HZSM-5(38), 10%CaO/HZSM-5(38), and 20%CaO/HZSM-5(38) catalysts. The HZSM-5(38), 5%CaO/HZSM-5(38), and 10%CaO/HZSM-5(38) catalysts showed type I isotherms, indicating the existence of microporosity [22].

After CaO modification, the samples had small hysteresis loops and poor porosity. The textural properties of the catalysts are summarized in Table 1. The BET surface area of HZSM-5 decreased significantly when the loading Ca increased from 5% to 10%. The specific surface areas of 5%CaO/HZSM-5(38) and 10%CaO/HZSM-5(38) were 274.37 m^2^/g and 198.39 m^2^/g, respectively, while their pore volumes were similar to those obtained in the original HZSM-5. However, the 20%CaO/HZSM-5(38) catalyst had an even lower specific surface area than the others, and the micropore structure of the 20%CaO/HZSM-5(38) catalyst was destroyed, as shown in Figure 2.

**Figure 3:**
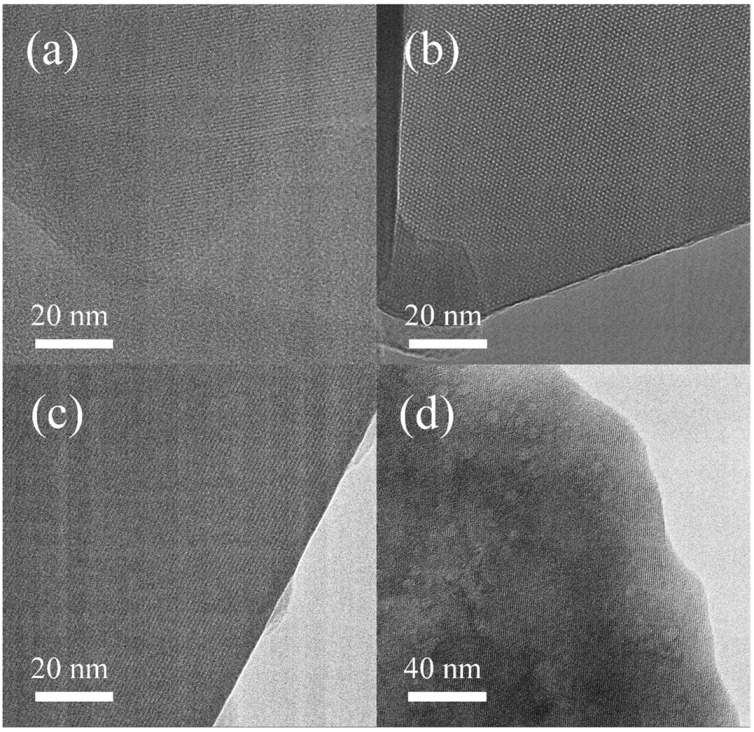
High-resolution scanning transmission electron microscopy (HR-STEM) image: (**a**) HZSM-5(38), (**b**) 5%CaO/HZSM-5(38), (**c**) 10%CaO/HZSM-5(38) and (**d**) 20%CaO/HZSM-5(38) catalysts.

The microporosity of HZSM-5(38), 5%CaO/HZSM-5(38), 10%CaO/HZSM-5(38), and 20%CaO/HZSM-5(38) catalysts were investigated by high-resolution scanning transmission electron microscopy (HR-STEM), as shown in Figure 3. It is obvious that the pore structures of the 5%CaO/HZSM-5(38) and 10%CaO/HZSM-5(38) samples were comparable to that of the HZSM-5(38) (Figure 3a–c), while higher loading of impregnated CaO was detrimental for the zeolite structures. The structure of the 20%CaO/HZSM-5(38) catalyst was broken (Figure 3d); this was consistent with the XRD results, which showed that the intensity of crystallinity had significantly decreased.

**Figure 4:**
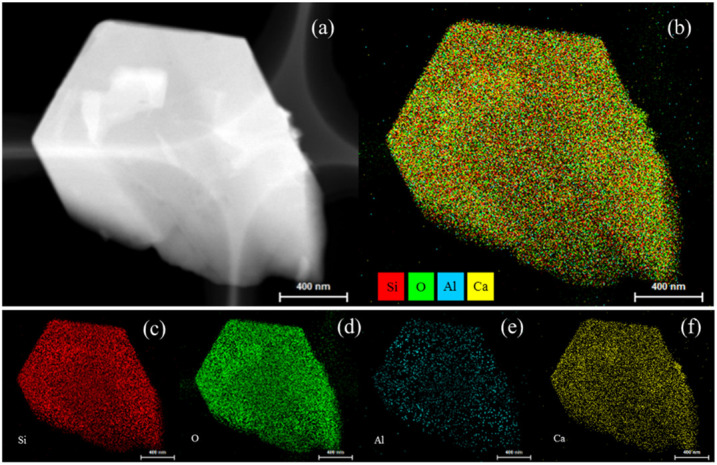
(**a**) High-angle annular dark-field scanning transmission electron microscope (HAADF-STEM) image, (**b**) STEM-EDS layered image, and (**c**–**f**) STEM-EDS map for Si, O, Al and Ca of the 10%CaO/HZSM-5(38) catalyst.

The EDS signals of the 10%CaO/HZSM-5(38) catalyst showed a high concentration of silicon and aluminum in the zeolite phase (Figure 4). In addition, the calcium species were found to be uniformly dispersed on the HZSM-5 zeolite of 10%CaO/HZSM-5(38), as shown in Figure 4. This result is in accordance with the XRD data. There was no obvious diffraction peak of the Ca in the XRD pattern, which indicated that the Ca species were highly distributed on the HZSM-5.

The CO_2_ and NH_3_-TPD results for different amounts of CaO in the HZSM-5 catalysts revealed the acid and base properties, respectively. The quantified amount of acid and base sites is summarized in Table 1. The total amount of basic sites increased from 5 to 10 wt% with increasing CaO content, suggesting that impregnating CaO into HZSM-5 zeolite can enhance the basicity of the catalysts. However, the total basic amount of HZSM-5 modified with 20% CaO decreased, possibly due to a loss of crystallinity, porosity, and specific surface area. These results suggest that the properties of HZSM-5 zeolite correlated with the basic site of CaO dispersed on surface catalysts.

For the NH_3_-TPD results, the acidity was not significantly different between HZSM-5 and the Ca-modified HZSM-5. However, after the addition of CaO, the number of total acid sites increased with the increases of 5% and 10% CaO loading but decreased with a 20% increase of CaO loading. Thus, the introduction of CaO could not eliminate acidity but rather, redistributed the acid sites on the HZSM-5 catalysts [23]. In addition, the total acid sites of the 20%CaO/HZSM-5(38) catalyst also decreased because of the destroyed structure of HZSM-5 zeolite, as supported by BET and XRD analysis. Even though the number of acid sites on the catalyst surface was determined by NH_3_-TPD analysis, the strength of the acidity and the distinction between acid types, e.g., Brønsted (B) and Lewis (L) acid sites could not be determined. Thus, the pyridine FT-IR method was used to study the nature of the acid sites. 

All samples showed characteristic bands at about 1540 and 1450 cm^−1^, which were assigned to the pyridine molecules adsorbed by the B and L acid sites, respectively [24]. The adsorption amount of pyridine degassed at temperatures of 150, 250 and 350 °C corresponded to the proportion of weak, medium, and strong acid sites. As shown in Table 2, when CaO was introduced into HZSM-5, it significantly reduced the B acid amount of HZSM-5, whilst the L acid amount increased. The 10%CaO/HZSM-5 catalyst showed L/B ratio values of of 8.4 (weak), 55.1 (medium), and 145.3 (strong), respectively, indicating that the 10%CaO/HZSM-5 catalyst possessed very strong L acid sites. These findings indicate that the addition of CaO to HZSM-5 zeolites not only adjusted the total amount of basic and acidic sites but also regulated the acid types which, in turn, promoted the catalytic performance of HZSM-5 for the catalytic cracking of glycerol to methanol.

**Figure 5:**
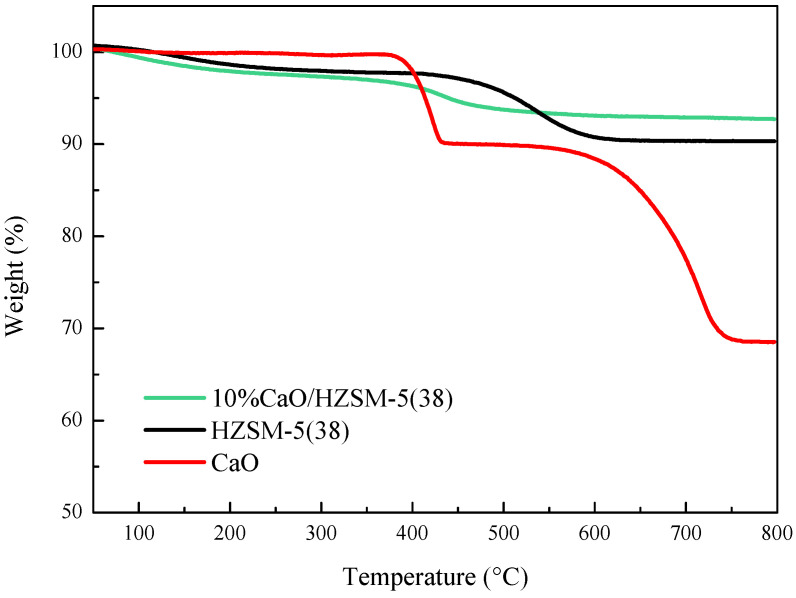
TGA profiles for the spent catalyst samples of 10%CaO/HZSM-5(38) and HZSM-5(38). (TOS = 2 h).

Figure 5 shows a thermo-gravimetric analysis (TGA) of the 10%CaO/HZSM-5(38), HZSM-5(38), and CaO catalysts after 2 h time-on-stream. A CaO event, carried out under N_2_ flow, was not perceptible after the catalytic test. The CaO catalyst is spent in two steps, i.e., the combustion of complex organic species of calcium and the decomposition of deposited carbonaceous material from glycerol or coke over the surface of CaO catalyst [25,26]. In the case of zeolite catalysts, the weight loss at lower temperature (<350 °C) was related to the removal of volatile compounds. Furthermore, the weight loss in a temperature range of 350–650 °C indicated the deposition of various types of carbonaceous species on the catalyst surface [27]. From the TGA result, the weight losses in the spent CaO, HZSM-5(38), and 10%CaO/HZSM-5(38) catalysts were about 32%, 10%, and 7%, respectively. The results show that the present HZSM-5(38) catalyst promoted by CaO displayed higher catalytic activity for methanol production and better coke resistance than the pure CaO and HZSM-5(38) catalysts.

### 3.2. Activity of the Bifunctional Catalyst in Glycerol Conversion to Methanol

**Figure 6:**
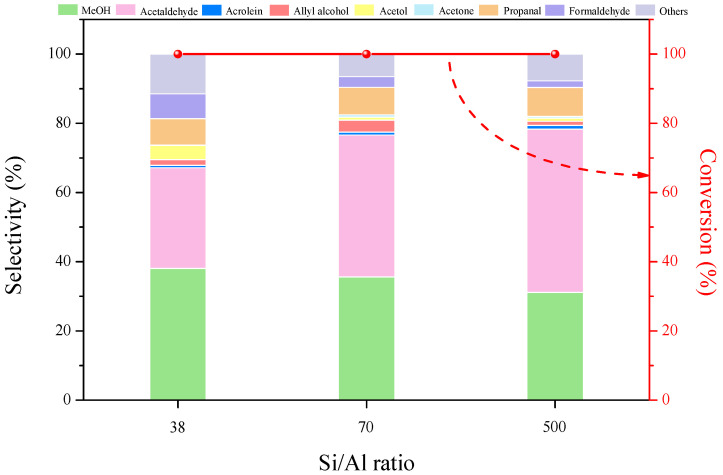
Glycerol conversion and products selectivity over 10%CaO/HZSM-5 catalysts with Si/Al ratio of 38, 70 and 500. Reaction conditions: T = 340 °C, feed flow = 0.03 mL min^−1^ (15 wt% glycerol/H_2_O), catalyst weight = 0.10 g, inert carrier = 20 mL min^−1^, TOS = 2 h.

Figure 6 shows the catalytic behavior of 10%CaO/HZSM-5 catalysts with Si/Al ratios of 38, 70, and 500 for the conversion of glycerol to methanol at 340 °C. The glycerol conversion was about 100%, but the methanol selectivity decreased when the Si/Al ratio increased. The 10%CaO/HZSM-5(38), 10%CaO/HZSM-5(70), and 10%CaO/HZSM-5(500) catalysts exhibited methanol selectivities of 38%, 36%, and 31%, respectively. Obviously, the increasing of Si/Al ratio over the catalysts is preferred to produce more acetaldehyde, as noted previously [28]. This result showed that methanol selectivity depends on the ratio of Si/Al, due to the difference in acid sites.

**Figure 7:**
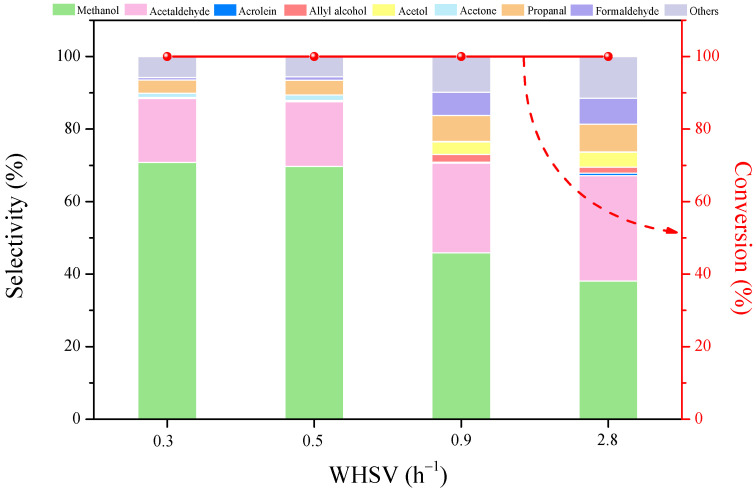
Glycerol conversion and products selectivity over 10%CaO/HZSM-5(38) catalysts at different WHSV. Reaction conditions: T = 340 °C, inert carrier = 20 mL min^−1^, TOS = 2 h.

The effect of weight hour space velocity (WHSV) on product distribution in glycerol conversion under reaction conditions of 340 °C, 1 bar, and N_2_ gas flow rate = 20 mL min^−1^ over CaO-promoted HZSM-5(38) catalysts is shown in Figure 7. It was found that the WHSV had a significant influence on the catalytic efficiency. It is interesting that the highest selectivity of methanol obtained at a range of WHSV = 0.3−0.5 h^−1^ was about 70%, with glycerol conversion of 100%. While enhancing WHSV from 0.5 to 0.9 and 2.8 h^−1^, the selectivity to methanol decreased but other products increased, such as formaldehyde, propanal, acetol, allyl alcohol, acrolein, etc. This result indicated that the dehydration of glycerol first produced other small products, such as acetol and acrolein [29], and that the dehydration of glycerol was much faster than the cracking of glycerol to methanol. It is thus suggested that the selectivity to methanol can improve with increased contact time.

**Figure 8:**
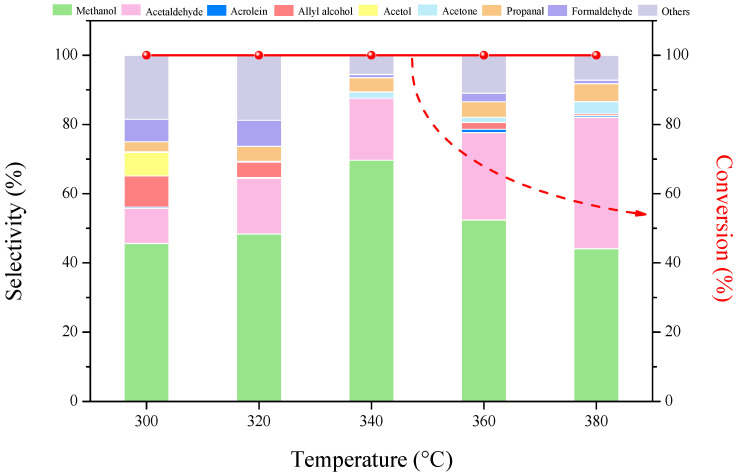
Glycerol conversion and product selectivity over 10%CaO/HZSM-5(38) catalysts at different temperatures. Reaction conditions: feed flow = 0.01 mL min^−1^ (15 wt% glycerol/H_2_O), catalyst weight = 0.20 g, inert carrier = 20 mL min^−1^, TOS = 2 h.

The influence of reaction temperature on product distribution in glycerol conversion over CaO-promoted HZSM-5(38) catalysts is shown in Figure 8. The catalytic performance of catalysts was investigated in the temperature range of 300 °C to 380 °C. The conversion of glycerol reached 100% in all cases, and the selectivity to methanol increased when the reaction temperature increased to 340 °C (70%); however, methanol selectivity did not increase further, even the reaction temperature was raised from 340 °C to 380 °C.

**Figure 9:**
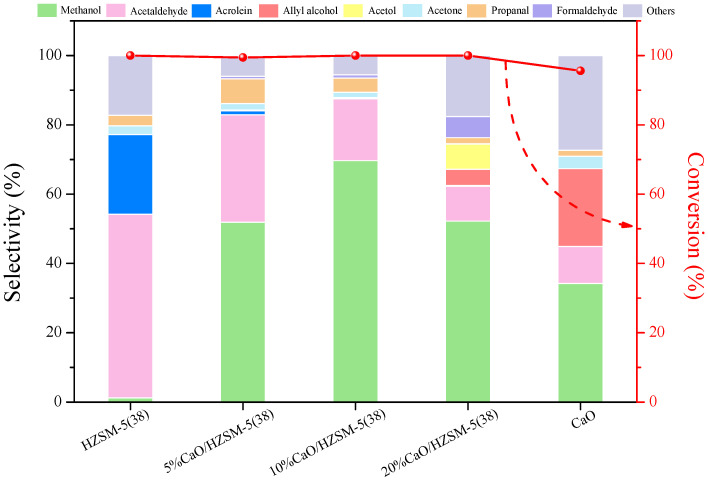
Glycerol conversion and products selectivity over HZSM-5(38), 5%CaO/HZSM-5(38), 10%CaO/HZSM-5(38), 20%CaO/HZSM-5(38), and CaO catalysts. Reaction conditions: T = 340 °C, feed flow = 0.01 mL min^−1^ (15 wt% glycerol/H_2_O), catalyst weight = 0.20 g, inert carrier = 20 mL min^−1^, TOS = 2 h.

Figure 9 shows the conversion of glycerol and the selectivities for glycerol dehydration to liquid products over the CaO/HZSM-5(38) catalysts with different amounts of CaO at 340 °C. The catalysts based on HZSM-5 showed glycerol conversion of 100% with time on stream. It is noteworthy that the 10%CaO/HZSM-5(38) catalyst showed the highest selectivity to methanol (70%). The high amounts of CaO doped into HZSM-5(38) led to low selectivity to methanol but increased the selectivity to other products, including formaldehyde, acetol, allyl alcohol, etc. This result shows that methanol selectivity depends on the properties of catalysts, such as their total acidity, total basicity, and pore structure. It is interesting to note that, based on the CO_2_-TPD results, the total basicity increased with 10 wt% loading amount of CaO, which showed the highest selectivity to methanol. According to the literature, the basic catalysts may be responsible for increasing the selectivity of methanol [5]. These results indicate that the synergistic effect of the combination of CaO and HZSM-5(38) enhances activity and selectivity to methanol.

Table S1 shows a comparison of the catalytic performance of 10%CaO/H-ZSM-5(38) with other catalysts for the synthesis of methanol from glycerol [5,26,30,31,32]. The 10%CaO/H-ZSM-5(38) catalyst performed best, both in terms of conversion and selectivity to methanol. We believe that this excellent catalytic performance is due to the bifunctional properties of the CaO modified HZSM-5 zeolite catalyst, which acts as both an acid and a base catalyst. Biodiesel fuel is produced from vegetable oil and methanol. Therefore, the transformation of glycerol to methanol would be highly desirable in the process of recycling, as shown in Figure S1.

## 4. Conclusions

In this paper, CaO modified HZSM-5 zeolite catalysts were successfully prepared, characterized, and tested in a reaction for glycerol conversion to methanol. The introduction of a CaO loading amount of 10 wt% resulted in an increase of Lewis acid sites and total basic sites, as measured by pyridine FT-IR and CO_2_-TPD, respectively. Moreover, the textural and catalytic properties of zeolite HZSM-5 for the cracking of glycerol were not destroyed with a loading amount of 10 wt% CaO, as characterized by XRD, BET, and NH_3_-TPD analysis. The results of this study indicate that high conversion of glycerol to methanol occurs with a suitable amount of HZSM-5 and CaO for the preservation of the properties of zeolite and basic catalysts. Moreover, the catalytic performance of the 10%CaO/HZSM-5 catalyst reached a maximum under optimal conditions, i.e., a WHSV of about 0.5 h^−1^, a reaction temperature of 340 °C, and atmospheric pressure. The 10%CaO/HZSM-5(38) catalyst showed excellent catalytic performance, with 70% selectivity to methanol and 100% conversion of glycerol. Thus, the obtained 10%CaO/HZSM-5(38) catalyst, with a high density of Lewis acid sites, high total acidity, and a larger external surface area showed superior catalytic performance in a synergistic catalysis system for the conversion of glycerol to methanol.

## Figures and Tables

**Table 1:** Structural parameters of fresh catalysts.

Catalyst	A_BET_ (m^2^ g^−1^)	Pore Volume v/(cm^3^ g^−^^1^) ^a^	Total Basicity (mmol g^−1^) ^b^	Total Acidity (mmol g^−1^) ^c^
HZSM-5(38)	388.02	0.18	1.22	0.88
5%CaO/HZSM-5(38)	274.37	0.13	1.53	1.02
10%CaO/HZSM-5(38)	198.39	0.10	1.77	1.02
20%CaO/HZSM-5(38)	22.25	0.01	1.28	0.71

^a^ BJH adsorption pore volume; ^b^ Total amount of absorption CO_2_ per gram catalyst; ^c^ Total amount of absorption NH_3_ per gram catalyst.

**Table 2:** Density of B and L acid sites in the 10%CaO/HZSM-5, CaO and HZSM-5 samples of pyridine, as determined by FT-IR.

Catalyst	Amount of Acid Sites (mmol g^−1^) (150 °C)				Amount of Acid Sites (mmol g^−1^) (250 °C)				Amount of Acid Sites (mmol g^−1^) (350 °C)			
	L	B	L+B	L/B	L	B	L+B	L/B	L	B	L+B	L/B
HZSM-5(38)	3.34	165.4	168.7	0.02	2.9	165.2	168.2	0.02	2.7	164.2	167.0	0.02
10%CaO/HZSM-5(38)	157.0	18.7	175.7	8.4	143.3	2.6	145.9	55.1	87.2	0.6	87.8	145.3

## Data Availability

Not applicable.

## Supplementary Materials

The following supporting information can be downloaded at: https://www.mdpi.com/article/10.3390/molecules27217221/s1, Figure S1: The scheme of selective conversion of glycerol to methanol over CaO-modified HZSM-5 Zeolite; Table S1: Comparison of the results in the production of methanol by various heterogeneous catalysts.
