# Supplementary material for: Selective Conversion of Glycerol to Methanol over CaO-Modified HZSM-5 Zeolite
Source: Molecules. 2022 Oct 25;27(21):7221. doi: 10.3390/molecules27217221 (PMC9655649; doi:10.3390/molecules27217221)
Supplement: Supplementary file 1 [file molecules-27-07221-s001.zip › molecules-1960115-supplementary.pdf]

Supporting Information

Selective Conversion of Glycerol to Methanol over CaO-Modified HZSM-5 Zeolite

Thachapan Atchimarungsri, [1,2] Xinhua Gao, [1,*] Kangzhou Wang, [1] Qingxiang Ma, [1] Jianli Zhang, [1] Subing Fan, [1] Fugui He, [1] Jumei Tian, [3] Prasert Reubroycharoen, [2,*] Tiansheng Zhao [1,*]

1 State Key Laboratory of High-efficiency Utilization of Coal and Green Chemical Engineering, College of Chemistry & Chemical Engineering, Ningxia University, Yinchuan 750021, P R China

2 Department of Chemical Technology, Faculty of Science, Chulalongkorn University, Bangkok 10330, Thailand

3 Ningxia Academy of Metrology & Quality Inspection, National Quality Supervision and Inspection Center for Coal and Coal Chemical Products (Ningxia), Yinchuan 750200, P R China

* Correspondence: gxh@nxu.edu.cn (X.G.); prasert.r@chula.ac.th (P.R.); zhaots@nxu.edu.cn (T.Z.)

Number of Pages: 3

Number of Figures: 1

Number of Table: 1

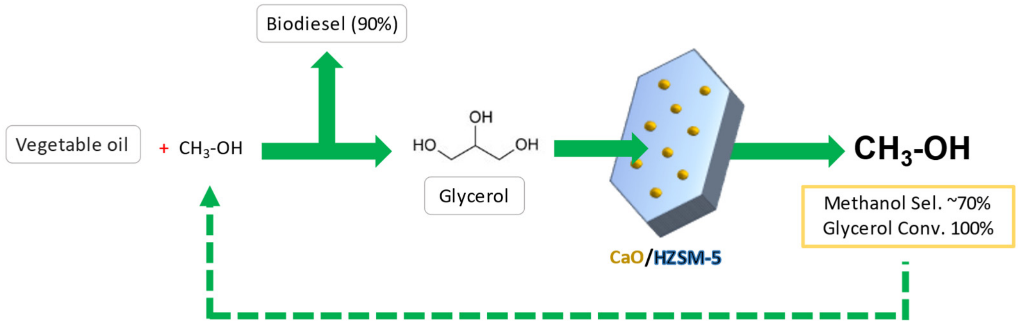

Figure S1. The scheme of selective conversion of glycerol to methanol over CaO-modified HZSM-5 Zeolite.

Table S1. Comparison of the results in the production of methanol by various heterogeneous catalysts.

Catalyst	Reaction conditions	Glycerol Conv. (%)	MeOH Selectivity (%)	Ref.
CaO	P = 1 bar, T = 320 °C, 10% glycerol aqueous solution 0.016 mL min^{-1}, carrier gas (N_2) 100 mL min^{-1}, 0.5 g catalyst, fixed-bed reactor	38%	57% (TOS = 3 h)	[1]
Ag/CaO-SiO_2	P = 1 bar, T = 280 °C, 5 wt% glycerol aqueous solution 0.06 mL min^{-1}, carrier gas (N_2), 0.1 g catalyst, fixed-bed reactor	~35%	~35%	[2]
Cu/HZSM-5 (Si/Al = 30)	P = 1 bar, T = 500 °C, Glycerol in a three neck round bottomed flask, carrier gas (N_2), fixed-bed reactor	100%	6.7% (TOS = 4 h)	[3]
γ-Al_2O_3	P = 1 bar, temperature of sonication = 40 °C, 10 wt% glycerol aqueous solution, 3% catalyst of glycerol (w/w), batch reactor equipped with an ultrasonic wave generator.	63.21 %	28.75% (TOS = 1.5 h)	[4]
Ni-V/γ-Al_2O_3	P = 1 bar, T = 500 °C, glycerol aqueous solution 0.083 mL min^{-1}, carrier gas (N_2) 25 mL min^{-1}, 0.085 g catalyst, fixed-bed reactor	99%	19% (TOS = 1 h)	[5]
10%CaO/HZSM-5 (38)	P = 1 bar, T = 340 °C, glycerol aqueous solution 0.01 mL min^{-1}, carrier gas (N_2) 20 mL min^{-1}, 0.2 g catalyst, fixed-bed reactor	100%	69.7% (TOS = 2 h)	This work

References

1. Haider, M.H.; Dummer, N.F.; Knight, D.W.; Jenkins, R.L.; Howard, M.; Moulijn, J.; Taylor, S.H.; Hutchings, G.J. Efficient green methanol synthesis from glycerol. *Nat. Chem.* **2015**, *7*, 1028–1032.

2. José de Ribamar, M.C.; Santos, R.C.; Coutinho, L.P.; Silva, O.R.; Barros, H.O.; Freire, V.N.; Valentini, A. CO_2 role on the glycerol conversion over catalyst containing CaO-SiO_2 doped with Ag and Pt. *Catal. Today*. **2020**, *344*, 199–211.

3. Mohamed, M.; T. Sie, T.; Amin, N.A.S.; Abdullah, T.A.T.; Mat, R. Conversion of glycerol to methanol in the presence of zeolite based catalysts. *2011 IEEE Conference on Clean Energy and Technology (CET)*. **2011**, 389-393.

4. Ruslan, K.; Sumarno, S.; Mahfud, M. The influence of using sonicator type to produce alcohol in the glycerol degradation process. *AIP Conference Proceedings*. **2017**, *1840*, 040004.

5. Ricardo, V.L.; Guilherme, K.M.; Anderson, T.; José, H.A.; Lucas da, S.R.; Bruno da, S.N.; Marcia, T.E.; Antoninho, V.; Luiz, F.D.P.; Neftali, L.V.C. Vanadium effect over γ-Al_2O_3-supported Ni catalysts for valorization of glycerol. *Fuel Process. Technol.* **2021**, *216*, 106773.
